# Where are the chiropractic clinical outcomes registries? A scoping review

**DOI:** 10.1186/s12998-025-00583-2

**Published:** 2025-05-25

**Authors:** Joel Carmichael, Kent Stuber, Katherine A. Pohlman, Amy Ferguson, Michele Maiers

**Affiliations:** 1 Department of Physical Medicine and Rehabilitation, School of Medicine, University of Colorado Anschutz Medical Campus, 13121 E 17th Ave, Mail Stop C244, Aurora, CO 80045 USA; 2 School of Chiropractic, Universidad Central del Caribe, P.O. Box 60327, Bayamón, 00960-6032 Puerto Rico; 3 Parker Research Center, Parker University, 2540 Walnut Hill Lane, Dallas, TX 75229 USA; 4 Department of Research and Innovation, Canadian Memorial Chiropractic College, 6100 Leslie St, Toronto, ON M2H 3J1 Canada; 5 Evidence Search Lab, 2008 Fairmeadow Drive, Richardson, TX 75080 USA; 6 Center for Research and Innovation, Northwestern Health Sciences University, 2501 W. 84th Street, Bloomington, MN 55431 USA; 7 RAND Research Across Complementary and Integrative Health Institutions (REACH) Center, RAND, 1776 Main Street, Santa Monica, CA 90401-2138 USA; 8 Lone Tree, USA

**Keywords:** Registry, Chiropractic clinical outcomes, Patient-reported outcome measures

## Abstract

**Objective:**

This scoping review maps chiropractic-specific clinical outcomes registries.

**Introduction:**

Clinical outcomes registries track patient outcomes to improve evidence-based practice and quality of care; however, their role in chiropractic remains unclear.

**Methods:**

This research adhered to Joanna Briggs Institute’s scoping review outline and methodology, as well as the PRISMA-ScR guidelines. Five databases were searched on January 9, 2025, with subsequent search of grey literature and citation tracking. Sources were included if they described chiropractic-specific registries that reported clinical outcomes data. Two reviewers independently screened 604 citations, extracting data into Excel. Variables included registry characteristics and clinical outcomes collected.

**Results:**

Only one dedicated chiropractic clinical outcomes registry was identified: Spine IQ, launched in 2016 in the US with approximately 50 chiropractors submitting data on over 2000 low back pain patients. Spine IQ collected patient-reported outcome measures including the Oswestry Disability Index, Bournemouth Questionnaire, and the PROMIS physical function measure. By 2018, Spine IQ had completed its pilot phase and planned expansion to 100 clinics. Three sources were excluded: one spine registry not collecting chiropractic outcomes and two databases that included chiropractic data in publications but did not qualify as registries.

**Conclusions:**

This review identified only Spine IQ as a dedicated chiropractic clinical outcomes registry, revealing a significant gap in registry infrastructure within the profession globally. The profession should explore the development of registries to enhance care quality, societal impact, and opportunities for collaborative research.

**Supplementary Information:**

The online version contains supplementary material available at 10.1186/s12998-025-00583-2.

## Introduction

A *clinical registry* is an organized data collection system used by clinicians [1, 2] to systematically gather clinical outcomes as data points over time from patients who share a specific health condition, intervention, or other characteristics. These clinical data points are relevant to changes in the health condition under study that may be associated with the intervention [3]. A registry’s primary purpose is to track trends in clinical outcomes, improve quality of care, benchmark healthcare performance, and support research and policymaking.

For the purposes of this scoping review, *chiropractic clinical outcomes* are defined as patient-reported clinical measures during or after chiropractic care, including patient symptoms and function [4]. These outcomes in a clinical registry are serially collected to gather data relevant to the condition or pathology under study.

For clarity, it is essential to distinguish clinical registries from clinical databases and Practice-based Research Networks (PBRNs). *Clinical databases* are broader and usually more comprehensive in scope whereas registries tend to be more focused and condition-specific [5, 6]. Clinical databases collect comprehensive patient data for more general purposes, such as patient billing and cost, administrative tasks, patient management (e.g., electronic health records), or ad hoc research. Data collection and the use of research in clinical databases are improvised or created as needed rather than being systematic or pre-planned, as is the case with clinical registries. Also, by contrast, *PBRNs* are organized collaborations of healthcare clinicians and practices that work together to answer healthcare questions, conduct research in everyday practice settings, and focus on care delivery and implementation science [7]. Unlike clinical registries, which use a standardized collection of clinical outcomes data for a pre-specified condition or set of conditions, PBRNs collect data for both prospective interventional trials and implementation research. While PBRNs may utilize clinical registry data collection modalities, they may also employ a broader range of data sources.

### Rationale

Clinical outcomes data are fundamental to answering important research questions as they form the foundation of evidence-based practice [8], support quality improvement and patient safety initiatives [9, 10], enhance patient-centered care [11], facilitate regulatory approval [12], inform health policy [13], and advance research methodologies [14]. Clinical registries amass large datasets that are cumulative over time, resulting in large sample sizes available for observational studies and pragmatic clinical research questions. By leveraging clinical outcomes data, clinicians and researchers can collaborate to enhance patient care and improve health outcomes [15, 16].

When systematically collected and aggregated over the long term, clinical outcomes data in registries can provide powerful, actionable information with greater feasibility, cost-effectiveness, and longer-term follow-up compared to time-limited, often resource-intensive clinical trials [17], enabling clinicians to refine clinical care pathways, optimize treatment outcomes over the longer term, and personalize care for individual patients [16]. Over the past 30 years [2] such registries have also been used by clinicians, researchers, industry leaders, and policymakers to enhance patient care and quality of life [18], update clinical care guidelines, and inform healthcare policy [19]. The adoption of automated technologies, such as smartphones and portable computing devices, has facilitated the rapid expansion of clinical outcomes registries across various healthcare disciplines worldwide [20–23]. These advancements have yielded impactful clinical research derived from real-world healthcare settings [20, 24, 25]. When applied with proper analytic techniques, registry studies can approach the methodological rigor of randomized controlled trials [26]. Furthermore, registries serve as valuable resources for training researchers and clinical education [27].

Chiropractic is a global profession with practitioners now established in over 90 countries across 6 continents [28]. As the profession continues to integrate into mainstream healthcare systems worldwide, the need for robust clinical evidence to inform best practices becomes increasingly important. Clinical outcomes registries may represent a valuable opportunity for the global chiropractic community to systematically document patient care and outcomes in diverse healthcare settings and cultural contexts.

### Objective

Given the value of clinical registries in several key professional domains, knowledge of existing chiropractic registries is essential. The objective of this scoping review is to map chiropractic clinical outcomes registries worldwide.

## Methods

### Protocol and registration

This scoping review was guided by the Joanna Briggs Institute (JBI) Manual for Evidence Synthesis methodology [29] and the Preferred Reporting Items for Systematic Reviews and Meta-Analyses Extension for Scoping Reviews (PRISMA-ScR) [30]. The objectives, inclusion criteria, and methodology for this scoping review were developed in accordance with Peters et al. [31] and prospectively registered and published online with INPLASY on June 20, 2023 (10.37766/inplasy2023.6.0064) [32]. This review did not require institutional review board (IRB) approval.

### Eligibility criteria

#### Types of participants

Eligible clinical registries for this review included those that described chiropractic patients of any age with any diagnosis, whose clinical outcomes data were collected and retained within the registry.

#### Concept

The core concept of this scoping review was to identify and map chiropractic-specific clinical registries reporting clinical outcomes collected in conjunction with chiropractic treatment encounters.

#### Context

This scoping review considered all clinical settings where clinical outcomes from chiropractic encounters were entered into a clinical registry.

### Eligible information sources

Eligible clinical registries collected clinical outcomes data from patients receiving chiropractic care. Information sources included scientific journals, grey literature (e.g., chiropractic trade journals and magazines), conference proceedings, and clinical practice guidelines. The search had no restrictions regarding socioeconomic status, healthcare system, location, demographics (including age, sex, gender, and race), military or civilian status, activity level, or insurance status of individuals whose data were collected by registries. Studies were excluded if they (1) did not meet registry definition criteria or (2) lacked clinical outcomes data from patients receiving chiropractic care.

### Search

A health sciences librarian collaborated with our research team to develop the search strategy and conduct a preliminary search. To ensure rigor and comprehensiveness, a second librarian independently applied the structured Peer Review of Electronic Search Strategies (PRESS) methodology [33]. We refined our methods by: (1) establishing precise definitions and differentiating features between clinical outcomes registries and databases to ensure sources were limited to registries only, (2) implementing citation tracking to identify additional registry reports, and (3) expanding our scope beyond clinical journals to include grey literature. To ensure clarity in evidence source selection for this review we specified that “chiropractic clinical outcomes” excluded health service utilization, claims, survey, cost-effectiveness, and administrative data but did not exclude medication use. After these methodological refinements, the scoping review was conducted again in its entirety.

Medline (Ovid), CINAHL (EBSCO), Index to Chiropractic Literature (ICL), Alt HealthWatch (EBSCO), and SPORTDiscus (EBSCO) were searched on January 9, 2025. We performed citation tracking using CitationChaser [34]. All citations were retained in Covidence and exported to EndNote for use by the research team. The comprehensive search strings used in this scoping review appear in Appendix 1, Supplementary Materials.

### Selection of sources of evidence

Citations were uploaded into EndNote 20 (Clarivate Analytics, PA, USA) and subsequently imported into Covidence (Veritas Health Innovation, Melbourne, Australia) for deduplication and screening. Two reviewers (KS and JC) conducted a pilot test of our initial title/abstract screening process for full-text review. They independently evaluated seven randomly selected abstracts to refine the process and ensure fidelity. This pilot resulted in 100% agreement, exceeding the a priori threshold of 85% set by the authors. The two reviewers performed full-text evaluation to select articles for data extraction. Following selection, they conducted reference checking to identify additional potential evidence sources.

Disagreements between reviewers during full-text review were resolved through discussion and iterative refinement. A third reviewer from the study team was available for arbitration but not required. Similarly, after completing full-text reviews in Covidence independently, all disagreements regarding final article selection were resolved through discussion between the two reviewers.

### Data charting process

This scoping review extracted data from full-text publications using a systematic manual approach rather than an automated selection tool. Data were organized in Excel and Word tables using predefined variables specific to chiropractic clinical registries that aligned with the objectives of this review. Supplementary information regarding registry characteristics was obtained through direct correspondence with registry owners and/or data managers.

### Data items

Prespecified chiropractic registry variables extracted into Excel included each of the following: name and country of registry, date of registry inception together with years of operation and date of registry cessation, health conditions catalogued, treatment types and clinical outcomes tracked, and patient enrollment numbers for the registry. We also extracted the stated purpose of the registry, if any, and the status of the registry as of January 9, 2025.

### Reporting of results

The broad scope and mapping objective of this review precluded risk of bias and sensitivity analyses. Following JBI scoping review methodology, quality appraisal of individual studies was not performed or required. The nature of the extracted data precluded quantitative synthesis. The editing of this manuscript was assisted by Claude 3.7 Sonnet, a large language model (LLM) developed by Anthropic. The LLM was used to identify spelling, grammar, and redundancy errors, and to assist in reducing word count, while the authors made all content decisions, performed analyses, and drew interpretations. This use of AI assistance is disclosed in accordance with the principles of scientific transparency.

## Results

### Inclusion of sources of evidence

**Fig. 1:**
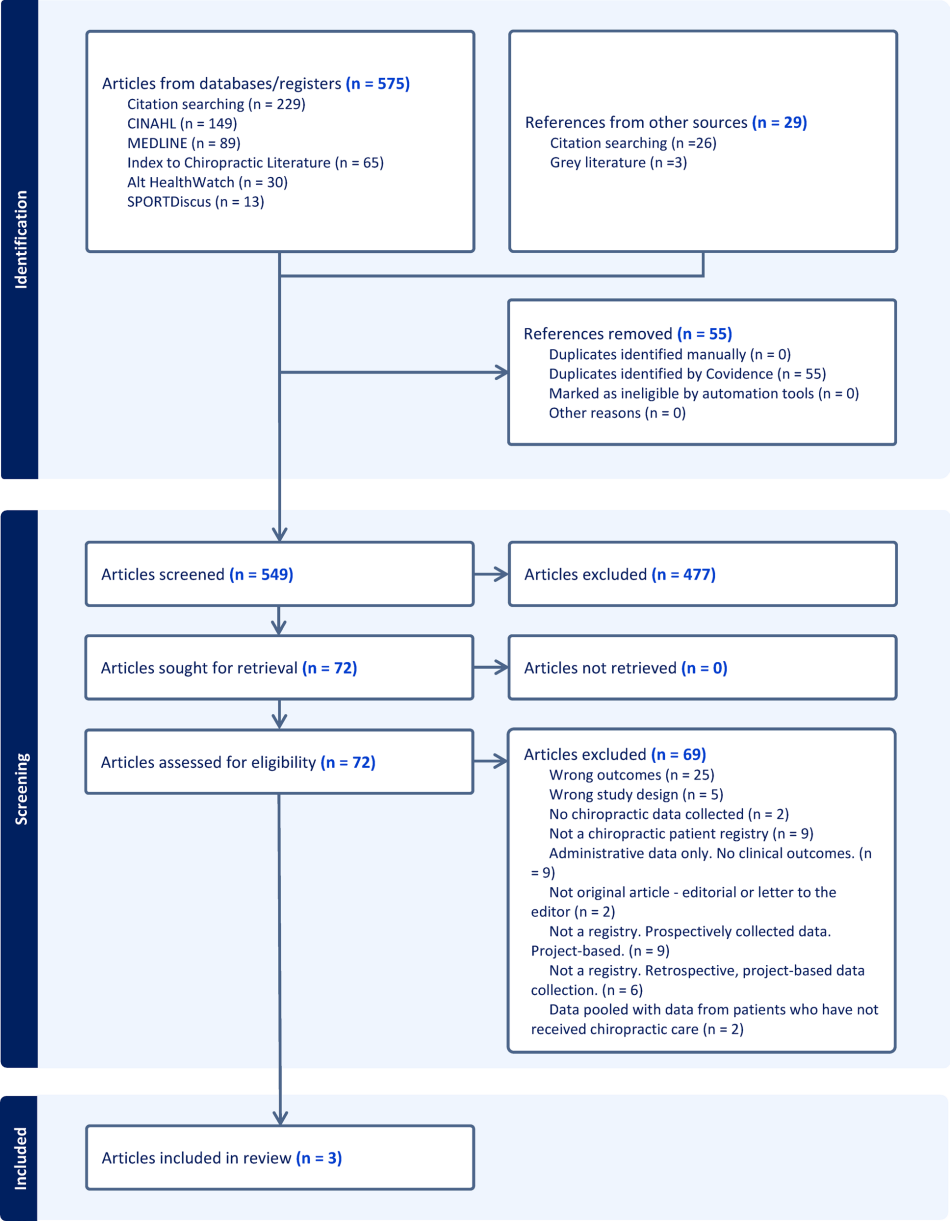
PRISMA flow diagram—chiropractic clinical outcomes registries

Our search strategy identified 604 evidence sources, with 549 remaining after deduplication in Covidence. Following title and abstract screening with refined definitions and methodology, 477 were excluded. Of the 72 full-text evidence sources assessed for eligibility, three met the inclusion criteria of being a clinical registry that collected chiropractic clinical outcomes data and were selected for data extraction (Fig. 1, PRISMA flow diagram). All three evidence sources described Spine IQ [1, 35, 36].

During our review, we also identified SpineData, a Danish clinical registry of people with low back pain of 2 to 12 months duration [37, 38]. However, after correspondence with the data manager, SpineData did not meet our inclusion criteria as it does not specifically collect outcomes data from chiropractic care, though it does collect spine-related outcomes from a regional non-surgical outpatient clinic. Although SpineData does not meet the inclusion criteria, we include a discussion of it as it represents a potential opportunity for integration with chiropractic data through its established infrastructure within the Danish healthcare system.

Citation tracking for Spine IQ revealed no publications, explicitly or indirectly, citing registry data.

### Characteristics of included studies

The clinical registry characteristics for Spine IQ are shown in Table 1.

**Table 1:** Chiropractic registry summary table

Registry name and country	Date of inception	Health conditions catalogued	Treatment types recorded	Clinical outcomes tracked	Years of operation	Patient enrollment numbers	Status	Purpose
Spine IQ™, USA (a non-profit organization)	July 2016	Spine conditions	Chiropractic treatment (not specified)	Systematic collection of Roland-Morris Disability Questionnaire, Oswestry Disability Index, Bournemouth Back Questionnaire and other Patient-Reported Outcome measures; functional status, pain levels	8 years	Approximately 100 baseline patients entered	Defunct	(1) Provide evidence of chiropractic effectiveness for patients with spine conditions (2) Support negotiations with payers by demonstrating cost-effectiveness and quality outcomes (3) Facilitate research

#### Spine IQ

Three abstracts were found in the grey literature that described Spine IQ, published in October 2016, January 2017, and December 2018, respectively [1, 35, 36]. At its launch in July 2016, Spine IQ began with a small cohort of approximately 50 chiropractors submitting data on over 2000 patients with low back pain. The initial registry focused specifically on outcomes related to back pain from chiropractic treatment. The articles suggest potential future expansion to include data from multiple non-surgical spine care disciplines, such as acupuncture and physical therapy.

Functional outcome measures collected by Spine IQ included the Oswestry Disability Index [39] and Bournemouth Questionnaire [40] for low back pain, and the PROMIS measure of functional status [41]—all with established property measurements and values for minimal clinically important difference (MCID).

The 2017 abstract emphasized using Spine IQ to “harness the power of big data.” The registry gained designation as a Qualified Clinical Data Registry (QCDR) from CMS in 2016, facilitating reimbursements through the Merit-based Incentive Payment System (MIPS) by gathering data via “easy-to-use electronic surveys.” By December 2018, Spine IQ had completed its pilot phase involving 10 chiropractic clinics representing approximately 50 chiropractors nationwide, proving the feasibility of its data collection and reporting systems, with plans to expand to 100 clinics in 2019.

#### SpineData

Though not meeting our inclusion criteria for a chiropractic clinical outcomes registry, SpineData warrants discussion as a model that could potentially incorporate chiropractic data in the future. SpineData is a clinical outcomes registry tracking patients with low back pain of 2 to 12 months duration the Spine Centre of Southern Denmark, a regional non-surgical outpatient facility at Kolding Hospital. The registry is administered through the University of Southern Denmark with funding from multiple sources, including private organizations (Industriens Arbejdsskadeforsikring and Trygfonden), a project grant from the Danish Foundation for Chiropractic Research and Postgraduate Education, and public funding from the Medical Department of the Spine Centre at Kolding Hospital.

Through personal correspondence with SpineData’s data manager we learned it was established in 2011 and launched operations in 2013, collecting clinical outcomes data on low back, mid-back, and neck pain until June 2021. In 2019 the mid-back and neck regions were combined into a single baseline questionnaire. By the end of May 2021, SpineData had collected 97,521 baseline questionnaires (77,669 complete, 7,104 partially complete, and 12,748 incomplete). SpineData was subsequently replaced by ‘Mine Rygdata’ (MiRD’), described as a ‘re-evaluated version of SpineData with slight modifications in content in structure,’ which collects outcomes data for two bodily regions: (1) low back, and (2) neck or mid-back, with comparable completion rates.

All excluded studies are listed in Appendix 2, Supplementary Materials, with their specific reasons for exclusion.

## Discussion

### Summary of key findings

This scoping review of chiropractic clinical outcomes registries identified Spine IQ in the U.S. as the profession’s only dedicated registry. The review highlights a significant gap in dedicated chiropractic outcomes registries compared to other healthcare disciplines, particularly orthopedics, which have successfully leveraged registries for research, quality improvement, and evidence-based practice for decades [2, 42].

### The impact of clinical outcomes registries in the chiropractic profession

The establishment of Spine IQ coincided with significant changes to the U.S. healthcare reimbursement landscape, particularly following the passage of the Medicare Access and CHIP Reauthorization Act of 2015 [43]. This legislation established the Merit-Based Incentive Payment System (MIPS) under the Centers for Medicare and Medicaid Services (CMS), which by 2024 had awarded approximately $500 million annually in performance-based bonuses to roughly 572,000 eligible clinicians. Dr. Christine Goertz, DC, PhD, founded Spine IQ with the strategic aim of helping chiropractors demonstrate superior clinical outcomes and potentially secure higher merit-based reimbursements. The registry accomplished this by implementing serial tracking of up to seven patient-reported outcome measures (PROs) that focused on care effectiveness rather than utilization. Spine IQ’s subsequent designation as a Qualified Clinical Data Registry (QCDR) was crucial as it enabled chiropractors to submit outcomes data directly to CMS’s merit-based incentive system. Despite this infrastructure and potential for financial benefit, chiropractic participation has remained nominal due to limited awareness and adoption within the profession. Currently only one domain—Rehabilitative Support for Musculoskeletal Care—remains open for chiropractic participation [43].

In contrast to the Spine IQ model in the U.S., SpineData in Denmark [37] represents an alternative approach, though it did not meet our inclusion criteria as a chiropractic-specific registry. Nevertheless, SpineData offers valuable insights as a potential model for future registry development due to its infrastructure and linkage capabilities within the Danish healthcare system. While it does not currently collect chiropractic clinical outcomes data, SpineData demonstrates the research potential of well-designed clinical outcomes registries. The substantial number of peer-reviewed publications citing SpineData [38, 44–58] illustrates how registry data can generate meaningful research output when adequately established and maintained. Additionally, SpineData’s evolution into MiRD exemplifies how registries can adapt to changing healthcare needs while preserving data continuity. For the chiropractic profession, SpineData represents a promising opportunity to integrate with established registry frameworks, particularly in countries with nationalized healthcare systems like Denmark where infrastructure for data collection and analysis already exists.

### Comparison with other healthcare registries

Since Brand et al. outlined the benefits of clinical registries in 1990 [2], many healthcare professions have successfully leveraged them to advance practice. Technological advances in mobile computing have enabled the automatic delivery of serial PRO questionnaires via email and SMS, allowing for substantial registry development. Modern electronic patient-reported outcomes collection (ePRO) systems have replaced earlier platforms, offering scalable solutions with improved follow-up data collection [59, 60].

The Multicenter Orthopaedic Outcomes Network (MOON) in the United States exemplifies this success, producing over 40 publications in its first decade [42]. MOON data directly influenced clinical practice by unnecessary interventions, validating specific rehabilitation approaches, and developing standardized rehabilitation protocols. By identifying modifiable outcome predictors that informed meaningful clinical trials, MOON demonstrates how registries can drive evidence-based practice evolution.

Similarly, national joint replacement registries track implant performance and patient outcomes, leading to continuous improvement in surgical techniques and device safety while providing surgeons with performance benchmarking [61]. While other healthcare disciplines have established numerous clinical registries across multiple countries [3, 18, 19, 24, 62–64], chiropractic has lagged in developing this critical research infrastructure.

### Comparison with large non-registry databases

Our review also identified non-registry databases that include chiropractic data, such as TriNetX®, a multi-hospital database, and the Danish national health registries [65, 66]. While these databases contain valuable information about chiropractic care utilization and costs, they fundamentally differ from clinical registries in that they lack systematic collection of clinical outcomes data. Furthermore, data from institutional sources like the U.S. Veterans Administration have limited generalizability to independent chiropractic practices—where most patient encounters occur—highlighting gaps that dedicated chiropractic clinical outcomes registries could address [65].

### Barriers to developing chiropractic clinical outcomes registries

Despite the clear benefits of clinical outcomes registries, implementing clinical registries in chiropractic faces numerous interconnected challenges. Financial constraints pose a significant barrier, as registries require substantial investment for infrastructure, data systems, training, and analysis, without the industry partnerships that fund similar efforts in other medical specialties. Technological challenges compound these issues, as many chiropractic practices lack necessary digital resources, while diverse electronic health records systems impede standardization. Regulatory compliance with privacy frameworks like HIPAA in the U.S. or the General Data Protection Regulation (GDPR) in Europe demands legal expertise beyond many organizations’ capabilities, though standardized de-identification procedures during routine care consent may simplify institutional review requirements. The profession’s diverse philosophies complicate consensus-building around standardized protocols, while immediate clinical concerns often overshadow long-term data collection priorities. Further challenges include establishing agreement on outcome measures, validated instruments, measurement frequency, and tracked conditions. Without meaningful incentives such as enhanced reimbursements, regulatory mandates, or professional recognition, practitioners remain hesitant to commit necessary resources. Finally, sustainability presents ongoing challenges, as exemplified by the SpineData to MiRD transition, where changes in funding, leadership, technology, and regulations necessitate continuous adaptation and resource commitment.

Future efforts to establish chiropractic clinical outcomes registries will need to address these challenges through collaborative approaches, sustainable funding models, and clear articulation of benefits to all stakeholders.

### Stakeholder perspectives on registry development

The development and implementation of chiropractic clinical outcomes registries involves multiple stakeholders, each with distinct perspectives, priorities, and concerns that must be considered for successful implementation. These stakeholders include chiropractors, patients, researchers, healthcare systems, policymakers, and educational institutions. Each group will bring differing perspectives on registries. Chiropractors may have concerns about workflow disruption and scrutiny while valuing the push toward evidence-based practice. Patients, as stakeholders, balance privacy concerns against strong interest in how their outcomes compare to “patients like me.” Researchers will weigh registry data access challenges against the opportunity to conduct comparative effectiveness studies and garner potential funding for innovative outcomes registry-based projects.

A stakeholder-centered approach that addresses the diverse perspectives and needs of these stakeholders through inclusive planning, clear communication about benefits and expectations, and meaningful engagement throughout the development process will enhance both the initial acceptance and long-term sustainability of chiropractic registries.

### Future directions for chiropractic registry development

Based on the findings of this review and the identified gaps, advancing chiropractic clinical registries requires a multifaceted approach including collaborative infrastructure development through partnerships between professional associations, academic institutions, and practice organizations, multi-stakeholder funding models, focused condition-specific registries, and standardized technical specifications. Leveraging technological advances is essential, utilizing mobile and cloud-based technologies, automated follow-up systems, artificial intelligence applications, and user-friendly dashboards that provide real-time feedback.

Strategic alignment with healthcare priorities demands designing registries that address contemporary challenges like non-pharmacological pain management, aligning outcome measures with other disciplines and approaches, enabling comparative clinical and cost-effectiveness analyses, and supporting quality reporting requirements. Building research capacity involves developing training programs, creating research fellowships, establishing governance structures, and prioritizing publication of findings. International collaboration requires coordinating across national boundaries to harmonize data elements, learning from existing registries, exploring comparative studies, and developing adaptable resources across care contexts and sub-populations. Patient engagement necessitates involving patients in registry design, developing explanatory materials, creating feedback mechanisms, and exploring efficient integration of patient-reported outcomes into workflows. Finally, incentive structures should advocate recognition in reimbursement models and credentialing, develop recognition programs, create clear feedback mechanisms demonstrating contributions to professional advancement, and explore certification programs for practices meeting quality benchmarks.

The development of robust chiropractic clinical outcomes registries represents a significant opportunity for the profession to strengthen its evidence base and credibility, demonstrate value within healthcare systems, and continuously improve quality of care. Strategic investment in this infrastructure would position chiropractic to make meaningful contributions to addressing major global public health challenges related to musculoskeletal pain and function.

### Limitations

Our review identified only one chiropractic registry (Spine IQ; U.S.) that met our inclusion criteria. Expanding our criteria to include databases, PBRNs, and other data repositories that capture data related to non-clinical aspects of chiropractic care may also be important to map, although outside the scope of this project.

The review did not conduct a quality assessment of included registries. However, it is worth noting that there are no universally adopted criteria for evaluating clinical outcomes registries, although diverse frameworks exist [6, 67].

Given the paucity of identified clinical registries, this review lacks comprehensive data on barriers to implementation and adoption specific to chiropractic registries. While we have proposed several barriers based on the literature from other healthcare disciplines [6, 68–70] direct evidence regarding chiropractic-specific challenges is limited.

While we have proposed a framework for considering stakeholder perspectives, this review did not directly capture the views of different stakeholders regarding registry development. Future qualitative research specifically addressing stakeholder perspectives would be valuable for informing implementation strategies.

## Conclusions

This scoping review of chiropractic clinical outcomes registries identified Spine IQ in the U.S. as the profession’s only registry. While other registries have the potential for integrating chiropractic data into established healthcare registries, a significant gap remains in dedicated registries within the chiropractic profession. Coordinated efforts among multiple stakeholders are necessary to inform the design and implementation of clinical registries dedicated to chiropractic clinical outcomes.

## Electronic supplementary material

Below is the link to the electronic supplementary material.

Supplementary Material 1

Supplementary Material 2

## Data Availability

No datasets were generated or analysed during the current study.
