# Supplementary material for: Where are the chiropractic clinical outcomes registries? A scoping review
Source: Chiropr Man Therap. 2025 May 25;33:22. doi: 10.1186/s12998-025-00583-2 (PMC12103756; doi:10.1186/s12998-025-00583-2)
Supplement: Supplementary file 1 — Supplementary Material 1 [file 12998_2025_583_MOESM1_ESM.docx]

# Appendix 1. All search strings used in this study

All databases were searched on January 9, 2025.

## **Medline (Ovid)**

Ovid MEDLINE(R) and Epub Ahead of Print, In-Process, In-Data-Review & Other Non-Indexed Citations, Daily and Versions <1946 to December 31, 2024>

1 Manipulation, Chiropractic/ or Manipulation, Orthopedic/ or Chiropractic/

2 Manipulation, Spinal/

3 chiropract*.mp.

4 1 or 2 or 3

5 exp Registries/

6 routinely collected health data/

7 exp Database Management Systems/

8 "regist*".ti,kf,kw.

9 (registry or registries).ab. not trial*.mp. [mp=title, book title, abstract, original title, name of substance word, subject heading word, floating sub-heading word, keyword heading word, organism supplementary concept word, protocol supplementary concept word, rare disease supplementary concept word, unique identifier, synonyms, population supplementary concept word, anatomy supplementary concept word]

10 "patient information".ab,ti.

11 "patient data".ab,ti.

12 data.ti.

13 5 or 6 or 7 or 8 or 9 or 10 or 11 or 12

14 4 and 13

## **CINAHL Complete (EBSCO)**

S1 (MH "Chiropractic") OR (MH "Manipulation, Chiropractic") OR (MH "Chiropractic Assessment") OR (MH "American Chiropractic Association") OR (MH "Students, Chiropractic") OR (MH "Research, Chiropractic") OR (MH "Education, Chiropractic") OR (MH "Chiropractic Practice") OR (MH "Chiropractors") OR (MH "Manipulation, Orthopedic")

S2 (MH "Manipulation, Orthopedic")

S3 spin* manipul*

S4 chiropract*

S5 (MH "Clinical Data Repository") OR (MH "Data Mining") OR (MH "Data Warehouse") OR (MH "Public Reporting of Healthcare Data") OR (MH "Routinely Collected Health Data")

S6 (TI registry OR registries) OR (AB registry OR registries)

S7 TI trial* OR AB trial*

S8 s6 NOT s7

S9 TI "patient data" OR AB "patient data"

S10 TI "patient information" OR AB "patient information"

S11 TI data

S12 S1 OR S2 OR S3 OR S4

S13 (S1 OR S2 OR S3 OR S4) AND (S5 OR S8 OR S9 OR S10 OR S11)

## **Alt HealthWatch (EBSCO)**

S1 DE "CHIROPRACTIC" OR DE "CHIROPRACTIC treatment for juvenile diseases" OR DE "CHIROPRACTIC treatment for infant diseases" OR DE "CHIROPRACTIC diagnosis" OR DE "RADIOGRAPHY in chiropractic" OR DE "CHIROPRACTIC records" OR DE "CRANIAL manipulation"

S2 DE "CRANIAL manipulation" OR DE "SPINAL adjustment"

S3 DE "CRANIOSACRAL therapy"

S4 chiropract*

S5 S1 OR S2 OR S3 OR S4

S6 TI ( registry or registries ) OR AB ( registry or registries )

S7 TI trial* OR AB trial*

S8 s6 NOT s7

S9 DE "MEDICAL registries"

S10 DE "DATA mining" OR DE "BIG data" OR DE "DATABASE management"

S11 TI "patient information" OR AB "patient information"

S12 TI "patient data" OR AB "patient data"

S13 TI data

S14 S8 OR S9 OR S10 OR S11 OR S12 OR S13

S15 S5 AND S14

## **SPORTDiscus with Full Text (EBSCO)**

S1 DE "CHIROPRACTIC" OR DE "SPINAL adjustment"

S2 DE "CRANIOSACRAL therapy"

S3 chiropract*

S4 S1 OR S2 OR S3

S5 TI ( registry OR registries ) OR AB ( registry OR registries )

S6 TI trial* OR AB trial*

S7 s5 NOT s6

S8 DE "MEDICAL registries"

S9 KW registry or registries

S10 TI "patient information" OR AB "patient information"

S11 TI "patient information" OR AB "patient information"

S12 TI "patient data" OR AB "patient data"

S13 TI data

S14 S7 OR S8 OR S9 OR S10 OR S11 OR S12 OR S13

S15 S4 AND S14

## **Index to Chiropractic Literature**

S1 Subject:\\\"Registries\\\"

S2 Subject:\\\"Electronic Health Records\\\"

S3 Article Title:registry OR Article Title:registries

S4 Article Title:trial OR Article Title:trials

S5 Article Title:registry OR Article Title:registries AND NOT Article Title:trial OR Article Title:trials

S6 Article Title:\"patient information\" OR Abstract/Notes:\"patient information\"

S7 Article Title:\"patient data\" OR Abstract/Notes:\"patient data\"

S8 Article Title:data

S9 Subject:\\\"Registries\\\" OR Subject:\\\"Electronic Health Records\\\" OR Article Title:registry OR Article Title:registries OR Article Title:trial OR Article Title:trials OR Article Title:registry OR Article Title:registries AND NOT Article Title:trial OR Article Title:trials OR Article Title:\"patient information\" OR Abstract/Notes:\"patient information\" OR Article Title:\"patient data\" OR Abstract/Notes:\"patient data\" OR Article Title:data
