# Supplementary material for: Where are the chiropractic clinical outcomes registries? A scoping review
Source: Chiropr Man Therap. 2025 May 25;33:22. doi: 10.1186/s12998-025-00583-2 (PMC12103756; doi:10.1186/s12998-025-00583-2)
Supplement: Supplementary file 2 — Supplementary Material 2 [file 12998_2025_583_MOESM2_ESM.docx]

APPENDIX 2 -- Retrieved publications that were excluded from data extraction

| Author(s) | Article Title | Citation | Reason for Exclusion |
| --- | --- | --- | --- |
| Hurwitz EL, Li D, Guillen J, et al. | Variations in Patterns of Utilization and Charges for the Care of Neck Pain in North Carolina, 2000 to 2009: A Statewide Claims' Data Analysis | Journal of Manipulative & Physiological Therapeutics 2016;39(4):240-51. DOI: 10.1016/j.jmpt.2016.02.007 | Wrong outcomes |
| Marrie RA, Hadjimichael O, Vollmer T | Predictors of alternative medicine use by multiple sclerosis patients | Multiple Sclerosis Oct 2003;9(5):461-6 | Wrong outcomes |
| Lalji R, Hofstetter L, Kongsted A, et al. | The Swiss chiropractic practice-based research network: a population-based cross-sectional study to inform future musculoskeletal research | Scientific Reports 2023;13(1):5655-NA. DOI: 10.1038/s41598-023-32437-3 | Wrong outcomes |
| Buser Z, Ortega B, D'Oro A, et al. | Spine Degenerative Conditions and Their Treatments: National Trends in the United States of America | Global Spine Journal 2017;8(1):57-67. DOI: 10.1177/2192568217696688 | No chiropractic data collected |
| Rosa J, Burke JR | Changes in Opioid Therapy Use by an Interprofessional Primary Care Team: A Descriptive Study of Opioid Prescription Data | Journal of Manipulative & Physiological Therapeutics 2021;44(3):186-195. DOI: 10.1016/j.jmpt.2021.01.003 | Wrong outcomes |
| Iachina M, Garvik OS, Ljungdalh PS, et al. | The clinical back pain courses described by information available in Danish central registries | BMC Health Services Research 2022;22(1):36-NA. DOI: 10.1186/s12913-021-07409-w | Data pooled with data from patients who have not received chiropractic care |
| da Silva T, Mills K, Kongsted A, et al. | What Is the Personal Impact of Recurrences of Low Back Pain? Subanalysis of an Inception Cohort Study | The Journal of Orthopaedic and Sports Physical Therapy 2020;50(6):294-300. DOI: 10.2519/jospt.2020.9345 | Not a chiropractic patient registry |
| Hartvigsen J, Davidsen M, Sogaard K, et al. | Self-reported musculoskeletal pain predicts long-term increase in general health care use: a population-based cohort study with 20-year follow-up | Scandinavian Journal of Public Health Nov 2014;42(7):698-704. DOI: 10.1177/1403494814542263 | Not a registry. Retrospective, project-based data collection. |
| Evans MW, Ndetan H, Hawk C | Use of chiropractic or osteopathic manipulation by adults aged 50 and older: An analysis of data from the 2007 National Health Interview Survey | Topics in Integrative Health Care 2010;1(2):Online access only 16 p | Wrong outcomes |
| Kania-Richmond A, Weeks L, Scholten J, et al. | Evaluating the feasibility of using online software to collect patient information in a chiropractic practice-based research network | Journal of the Canadian Chiropractic Association 2016;60(1):93-105 | Not a registry. Prospectively collected data. Project-based. |
| Lalji R, Muñoz Laguna J, Kauth J, et al. | What Gets Measured Gets Managed: A Scoping Review of Musculoskeletal Research Conducted Within Practice-Based Research Networks | American Journal of Physical Medicine & Rehabilitation 2024;103(9):e113-e121 | Wrong study design |
| Ndetan H, Evans MW, Hawk C, et al. | Chiropractic or Osteopathic Manipulation for Children in the United States: An Analysis of Data from the 2007 National Health Interview Survey | Journal of Alternative & Complementary Medicine 2012;18(4):347-353. DOI: 10.1089/acm.2011.0268 | Wrong outcomes |
| Young KJ, Fitzgerald J, Field J, et al. | A descriptive analysis of the contents of Care Response, an international data set of patient-reported outcomes for chiropractic patients | Chiropractic & Manual Therapies 2023;31(1):1-8. DOI: 10.1186/s12998-023-00509-w | Not a chiropractic patient registry |
| Trager RJ, Cupler ZA, Srinivasan R, et al. | Association between chiropractic spinal manipulation and gabapentin prescription in adults with radicular low back pain: retrospective cohort study using US data | BMJ Open 2023;13(7):e073258. DOI: 10.1136/bmjopen-2023-073258 | Administrative data only. No clinical outcomes. |
| Trager RJ, Gliedt JA, Labak CM, et al. | Association between spinal manipulative therapy and lumbar spine reoperation after discectomy: a retrospective cohort study | BMC Musculoskeletal Disorders 2024;25(1):46-NA. DOI: 10.1186/s12891-024-07166-x | Administrative data only. No clinical outcomes. |
| Nim CG, Kongsted A, Downie A, et al. | Temporal stability of self-reported visual back pain trajectories | PAIN 2022;163(11):e1104-e1114. DOI: 10.1097/j.pain.0000000000002661 | Not a registry. Prospectively collected data. Project-based. |
| Jensen RK, Ris I, Linnebjerg E, et al. | The utilisation of regulated standardised care packages by Danish chiropractors: a mixed methods study | Chiropractic & Manual Therapies 2022;30(1):1-13. DOI: 10.1186/s12998-022-00423-7 | Wrong outcomes |
| Hansen A, Morsø L, Stochkendahl MJ, et al. | Demographic and clinical characteristics of patients with low back pain in primary and secondary care settings in Southern Denmark | Scandinavian Journal of Primary Health Care 2023;41(2):152-159. DOI: 10.1080/02813432.2023.2196548 | Wrong outcomes |
| Sabatino MJ, Burroughs PJ, Moore HG, et al. | Spine coding transition from ICD-9 to ICD-10: Not taking advantage of the specificity of a more granular system | North American Spine Society Journal 2020;4(NA):100035-NA. DOI: 10.1016/j.xnsj.2020.100035 | Wrong outcomes |
| Belbeck J | Being data driven | Canadian Chiropractor 2018;23(6):4-4 | Not a chiropractic patient registry |
| Hawk C, Ndetan H, Evans MW | Potential role of complementary and alternative health care providers in chronic disease prevention and health promotion: an analysis of National Health Interview Survey data | Preventive Medicine Jan 2012;54(1):18-22. DOI: 10.1016/j.ypmed.2011.07.002 | Wrong outcomes |
| Hudson K, Cost B, Greenstein J | Unlocking the Future: Chiropractic Future Strategic Plan Data Lake Project | American Chiropractor 2024;46(8):16-16 | Not original article - editorial or letter to the editor |
| Smith M, Carber L | A compilation of chiropractic and complementary/alternative medicine (CAM) data from public-use national surveys: report on a health-services research resource for the chiropractic and CAM scientific community | Journal of Manipulative & Physiological Therapeutics 2002;25(9):573-578. DOI: 10.1067/mmt.2002.128366 | Wrong outcomes |
| Ekholm O, Hesse U, Davidsen M, et al. | The study design and characteristics of the Danish national health interview surveys | Scandinavian Journal of Public Health 2009;37(7):758-765. DOI: 10.1177/1403494809341095 | Wrong study design |
| Davis BA, Dunn AS, Golley DJ, et al. | Chiropractic Clinical Outcomes Among Older Adult Male Veterans With Chronic Lower Back Pain: A Retrospective Review of Quality-Assurance Data | Journal of Chiropractic Medicine 2022;21(2):77-82. DOI: 10.1016/j.jcm.2022.02.004 | Wrong outcomes |
| Licciardone JC, Pandya V | Use of Complementary Health Approaches for Chronic Low-Back Pain: A Pain Research Registry-Based Study | Journal of Alternative & Complementary Medicine 2020;26(5):369-375. DOI: 10.1089/acm.2019.0448 | Not a chiropractic patient registry |
| Morso L, Bogh SB, Ris I, et al. | Mind the gap - Evaluation of the promotion initiatives for implementation of the GLA:D R back clinician courses | Musculoskeletal Science & Practice 06 2021;53():102373. DOI: 10.1016/j.msksp.2021.102373 | Not a chiropractic patient registry |
| Haas M, Nyiendo J, Lloyd C, et al. | Data management in practice-based research | Journal of Manipulative & Physiological Therapy Jan 2002;25(1):49-57 | Not a chiropractic patient registry |
| Clausen S, Hartvigsen J, Johansson MS, et al. | Healthcare Utilisation in Danish Primary Care Among Patients With Low Back or Neck/Thoracic Spine Pain Before and After Assessment in Secondary Care | Musculoskeletal Care 2024;22(4):1-9. DOI: 10.1002/msc.70017 | Wrong outcomes |
| Raina P, Torrance-Rynard V, Wong M, et al. | Agreement between self-reported and routinely collected health-care utilization data among seniors | Health Services Research Jun 2002;37(3):751-74 | Wrong outcomes |
| Jensen MS, Olsen KR, Morso L, et al. | Does changed referral options affect the use of MRI for patients with low back pain? Evidence from a natural experiment using nationwide data | BMJ Open 06 27 2019;9(6):e025921. DOI: 10.1136/bmjopen-2018-025921 | No chiropractic data collected |
| Jensen RK, Jensen TS, Grøn S, et al. | Prevalence of MRI findings in the cervical spine in patients with persistent neck pain based on quantification of narrative MRI reports | Chiropractic & Manual Therapies 2019;27(1):N.PAG-N.PAG. DOI: 10.1186/s12998-019-0233-3 | Wrong outcomes |
| Lisi AJ, Brandt CA | Trends in the Use and Characteristics of Chiropractic Services in the Department of Veterans Affairs | Journal of Manipulative & Physiological Therapeutics 2016;39(5):381-386. DOI: 10.1016/j.jmpt.2016.04.005 | Wrong outcomes |
| Rosati LM, Vezzetti A, Redd KT, et al. | Early Anticoagulation or Antiplatelet Therapy Is Critical in Craniocervical Artery Dissection: Results from the COMPASS Registry | Cerebrovascular Diseases 2020;49(4):369-374. DOI: 10.1159/000509415 | Wrong outcomes |
| McCoy M, Blanks RH | Uncovering the global burden of vertebral subluxation: the need for centralized data repositories | Journal of Vertebral Subluxation Research (JVSR) 2006;():1-5 | Not original article - editorial or letter to the editor |
| Keeney BJ, Fulton-Kehoe D, Turner JA, et al. | Early predictors of lumbar spine surgery after occupational back injury: results from a prospective study of workers in Washington State | Spine 2013;38(11):953-964. DOI: 10.1097/brs.0b013e3182814ed5 | Wrong outcomes |
| Trager RJ, Nichols MD, Barnett TD, et al. | Impact of Integrative Health and Medicine on Costs Associated with Adult Health System Beneficiaries with Musculoskeletal Conditions: A Retrospective Cohort Study | Journal of Integrative and Complementary Medicine 2024;NA(NA):NA-NA. DOI: 10.1089/jicm.2023.0812 | Wrong outcomes |
| Nyiendo J, Haas M, Goldberg B, et al. | Patient characteristics and physicians' practice activities for patients with chronic low back pain: A practice-based study of primary care and chiropractic physicians | Journal of Manipulative and Physiological Therapeutics 2001;24(2):92-100. DOI: 10.1067/mmt.2001.112565 | Wrong study design |
| Herman PM, Edgington SE, Hurwitz EL, et al. | Predictors of visit frequency for patients using ongoing chiropractic care for chronic low back and chronic neck pain; analysis of observational data | BMC Musculoskeletal Disorders 2020;21(1):1-14. DOI: 10.1186/s12891-020-03330-1 | Not a registry. Prospectively collected data. Project-based. |
| Trager RJ, Daniels CJ, Perez JA, et al. | Association between chiropractic spinal manipulation and lumbar discectomy in adults with lumbar disc herniation and radiculopathy: retrospective cohort study using United States' data | BMJ Open 12 16 2022;12(12):e068262. DOI: 10.1136/bmjopen-2022-068262 | Not a registry. Retrospective, project-based data collection. |
| Lalji R, Hofstetter L, Kongsted A, et al. | The Swiss chiropractic practice-based research network: a population-based cross-sectional study of chiropractic clinicians and primary care clinics to inform future musculoskeletal health care research | NA 2022;NA(NA):NA-NA. DOI: 10.21203/rs.3.rs-2041025/v1 | Wrong outcomes |
| Grøn S, Jensen RK, Kongsted A | Beliefs about back pain and associations with clinical outcomes: a primary care cohort study | BMJ Open 2022;12(5):e060084-e060084. DOI: 10.1136/bmjopen-2021-060084 | Wrong outcomes |
| Simonsen GD, Jensen TS, Kongsted A | Reassuring Patients With Low Back Pain in Primary Care Consultations: Does it Happen, and Does it Matter? A ChiCo Cohort Study | The Clinical Journal of Pain 2021;37(8):598-606. DOI: 10.1097/ajp.0000000000000946 | Wrong outcomes |
| Young BA, Lisi AJ, Halloran SM, et al. | Assessing conditions seen and services provided by Veterans Health Administration chiropractors: Comparing provider self-report with electronic health record data | Journal of Contemporary Chiropractic 2021;4(1):83-91 | Administrative data only. No clinical outcomes. |
| Adams J, Steel A, Moore C, et al. | Establishing the ACORN national practitioner database: Strategies to recruit practitioners to a national practice-based research network | Journal of Manipulative & Physiological Therapy Oct 2016;39(8):594-602 | Not a chiropractic patient registry |
| Lalji R, Hofstetter L, Kongsted A, et al. | Swiss chiropractic practice-based research network and musculoskeletal pain cohort pilot study: protocol of a nationwide resource to advance musculoskeletal health services research | BMJ Open 2022;12(7):e059380-e059380. DOI: 10.1136/bmjopen-2021-059380 | Not a registry. Prospectively collected data. Project-based. |
| Kongsted A, Nielsen OL, Christensen HW, et al. | The Danish Chiropractic Low Back Pain Cohort (ChiCo): Description and Summary of an Available Data Source for Research Collaborations | Clinical Epidemiology 2020;12():1015-1027. DOI: 10.2147/CLEP.S266220 | Not a registry. Prospectively collected data. Project-based. |
| Gliedt JA, Anderson BR, Reynolds M, et al. | The prevalence of substance use disorders in chiropractic patients in the United States: a descriptive study of two national datasets | Journal of Substance Use 2023;NA(NA):1-5. DOI: 10.1080/14659891.2023.2293772 | Wrong study design |
| Amundsen O, Moger TA, Holte JH, et al. | Patient characteristics and healthcare use for high-cost patients with musculoskeletal disorders in Norway: a cohort study | BMC Health Services Research 2024;24(1):1583. DOI: 10.1186/s12913-024-12051-3 | Wrong study design |
| Lykkegaard CR, Wedderkopp N, Wehberg S, et al. | Does children's healthcare seeking change after participation in a musculoskeletal study? A register-based study | BMC Primary Care 2023;24(1):271. DOI: 10.1186/s12875-023-02233-z | Wrong outcomes |
| Trager RJ, Makineni PS, Williamson TJ | Patients With a History of Spine Surgery Receiving Chiropractic Spinal Manipulation in US Academic Health Centers: A Cross-Sectional Cohort Study | Cureus 2023;15(4):e37216-NA. DOI: 10.7759/cureus.37216 | Not a registry. Prospectively collected data. Project-based. |
| Trager RJ, Cupler ZA, Srinivasan R, et al. | Chiropractic spinal manipulation and likelihood of tramadol prescription in adults with radicular low back pain: a retrospective cohort study using US data | BMJ Open 2024;14(5):e078105. DOI: 10.1136/bmjopen-2023-078105 | Not a registry. Retrospective, project-based data collection. |
| Ndetan H, Hawk C, Sekhon VK, et al. | The Role of Chiropractic Care in the Treatment of Dizziness or Balance Disorders: Analysis of National Health Interview Survey Data | Journal of Evidence-Based Complementary & Alternative Medicine Apr 2016;21(2):138-42. DOI: 10.1177/2156587215604974 | Administrative data only. No clinical outcomes. |
| Trager RJ, Baumann AN, Perez JA, et al. | Association between chiropractic spinal manipulation and cauda equina syndrome in adults with low back pain: Retrospective cohort study of US academic health centers | PLoS One 2024;19(3):e0299159-e0299159. DOI: 10.1371/journal.pone.0299159 | Not a registry. Retrospective, project-based data collection. |
| Owens Jr EF, Esposito J, Hosek RS, et al. | Assessing the feasibility of using an electronic records database system in use in a group of private chiropractic clinics for practice-based research. / Évaluation des possibilités d'utilisation d'une base de données sur les dossiers électroniques par un groupe de cliniques de chiropratique privées aux fins de recherches fondées sur la pratique | Journal of the Canadian Chiropractic Association 2021;65(1):94-104 | Not a chiropractic patient registry |
| Kinge JM, Dieleman JL, Karlstad O, et al. | Disease-specific health spending by age, sex, and type of care in Norway: a national health registry study | BMC Medicine 06 06 2023;21(1):201. DOI: 10.1186/s12916-023-02896-6 | Data pooled with data from patients who have not received chiropractic care |
| Mose S, Kent P, Smith A, et al. | Trajectories of Musculoskeletal Healthcare Utilization of People with Chronic Musculoskeletal Pain - A Population-Based Cohort Study | Clinical Epidemiology 2021;13():825-843. DOI: 10.2147/CLEP.S323903 | Wrong outcomes |
| Hurwitz EL, Morgenstern H | The effect of comorbidity on care seeking for back problems in the United States | Annals of Epidemiology 1999;9(4):262-270. DOI: 10.1016/s1047-2797(98)00059-3 | Not a registry. Prospectively collected data. Project-based. |
| Amundsen O, Moger TA, Holte JH, et al. | Combination of health care service use and the relation to demographic and socioeconomic factors for patients with musculoskeletal disorders: a descriptive cohort study | BMC Health Services Research 2023;23(1):858. DOI: 10.1186/s12913-023-09852-3 | Not a registry. Prospectively collected data. Project-based. |
| Legorreta AP, Metz RD, Nelson CF, et al. | Comparative Analysis of Individuals With and Without Chiropractic Coverage Patient Characteristics, Utilization, and Costs | Archives of Internal Medicine 2004;164(18):1985-1992. DOI: 10.1001/archinte.164.18.1985 | Administrative data only. No clinical outcomes. |
| Whedon JM, Haldeman S, Petersen CL, et al. | Temporal Trends and Geographic Variations in the Supply of Clinicians Who Provide Spinal Manipulation to Medicare Beneficiaries: A Serial Cross-Sectional Study | Journal of Manipulative and Physiological Therapeutics 2021;44(3):177-185. DOI: 10.1016/j.jmpt.2021.02.002 | Administrative data only. No clinical outcomes. |
| Trager RJ, Anderson BR, Casselberry RM, et al. | Guideline-concordant utilization of magnetic resonance imaging in adults receiving chiropractic manipulative therapy vs other care for radicular low back pain: a retrospective cohort study | BMC Musculoskeletal Disorders 2022;23(1):554-NA. DOI: 10.1186/s12891-022-05462-y | Not a registry. Retrospective, project-based data collection. |
| Valvi N, Tamargo JA, Braithwaite D, et al. | Household Income Is Associated with Chronic Pain and High-Impact Chronic Pain among Cancer Survivors: A Cross-Sectional Study Using NHIS Data | Cancers 2024;16(16):2847. DOI: 10.3390/cancers16162847 | Not a registry. Retrospective, project-based data collection. |
| Sharma R, Haas M, Stano M | Patient attitudes, insurance, and other determinants of self-referral to medical and chiropractic physicians | American Journal of Public Health 2003;93(12):2111-2117. DOI: 10.2105/ajph.93.12.2111 | Wrong outcomes |
| ACA | ACA Your ADVOCATE. HHS Releases Medicare Payment and Procedure Data | ACA News (American Chiropractic Association) 2014;10(5):6-6 | Administrative data only. No clinical outcomes. |
| Lalji R, Hofstetter L, Kongsted A, et al. | Swiss chiropractic cohort (Swiss ChiCo) pilot study: feasibility for a musculoskeletal cohort study conducted within a nationwide practice-based research network | European Spine Journal: Official Publication of the European Spine Society, the European Spinal Deformity Society, and the European Section of the Cervical Spine Research Society 2024;33(5):2068-2078. DOI: 10.1007/s00586-024-08175-z | Not a registry. Prospectively collected data. Project-based. |
| Chevan J, Riddle DL | Factors associated with care seeking from physicians, physical therapists, or chiropractors by persons with spinal pain: a population-based study | The Journal of Orthopaedic and Sports Physical Therapy 2011;41(7):467-476. DOI: 10.2519/jospt.2011.3637 | Administrative data only. No clinical outcomes. |
| Weeks WB, Leininger B, Whedon JM, et al. | THE ASSOCIATION BETWEEN USE OF CHIROPRACTIC CARE AND COSTS OF CARE AMONG OLDER MEDICARE PATIENTS WITH CHRONIC LOW BACK PAIN AND MULTIPLE COMORBIDITIES | Journal of Manipulative and Physiological Therapeutics 2016;39(2):63-75. DOI: 10.1016/j.jmpt.2016.01.006 | Administrative data only. No clinical outcomes. |
| Kent P, Kongsted A, Jensen TS, Albert HB, Schiottz-Christensen B, Manniche C. | SpineData – A Danish clinical registry of people with chronic low back pain. | Clinical Epidemiology 2015; 7:369-80. | Not a chiropractic patient registry. |
